# Lower skeletal muscle index and early complications in patients undergoing radical cystectomy for bladder cancer

**DOI:** 10.1186/1477-7819-12-14

**Published:** 2014-01-14

**Authors:** Fangning Wan, Yao Zhu, Chengyuan Gu, Xudong Yao, Yijun Shen, Bo Dai, Shilin Zhang, Hailiang Zhang, Jingyi Cheng, Dingwei Ye

**Affiliations:** 1 Department of Urology, Fudan University Shanghai Cancer Center, No. 270 Dong’an Road, Shanghai 200032, People’s Republic of China; 2 Department of Oncology, Fudan University Shanghai Medical College, Shanghai, China; 3 Department of Nuclear Medicine, Fudan University Shanghai Cancer Center, Shanghai, China

**Keywords:** Bladder cancer, Radical cystectomy, SMI, Postoperative complications, Sarcopenia

## Abstract

**Background:**

Radical cystectomy (RC) is the standard treatment for patients with muscle-invasive bladder cancer (BC), and it is also a valid option for selected patients with high-risk non-muscle-invasive BC. The purpose of this study was to evaluate the effect on the lower skeletal muscle index (SMI) of short-term postoperative complications of radical cystectomy (RC) in patients with bladder cancer (BC).

**Methods:**

A total of 247 patients who received RC for BC and 204 age-matched healthy population-based controls were retrospectively assessed. SMI was measured by preoperative computed tomography scans at the L4 to L5 level. Early complications were graded by Clavien-Dindo classification; severity of grade III or greater was identified as a severe complication. Logistic regression was utilized to determine the relationships between covariables and severe complications.

**Results:**

A total of 125 (50.61%)/19 (7.69%) patients exhibited overall/severe complications during the early postoperative period. SMI was strongly associated with gender (*P* <0.01), but not age and body mass index (BMI), among patients with BC. Compared with the matched control group, BC patients exhibited lower SMI. The difference was statistically significant in the subgroup of male patients (*P* = 0.03). In the multivariate analysis, SMI was an independent predictor of developing severe complications. Each 1 cm^2^/m^2^ increase in SMI was associated with a decrease in the odds of morbidity by 4.8%.

**Conclusions:**

A lower SMI is frequently observed in bladder cancer patients undergoing RC and is shown to be strongly associated with early complications following surgery.

## Background

Radical cystectomy (RC) is the standard treatment for patients with muscle-invasive bladder cancer (BC), and it is also a valid option for selected patients with high-risk non-muscle-invasive BC. RC includes simultaneous surgery on the urinary tract, lymph nodes, and intestines; complications frequently occur after this extensive procedure. Using standardized reporting methods, early complication rates ranged from 49% to 64% in a recently reported patient series [1-3]. Therefore, identification of risk factors associated with complications following RC through individual patient counseling, perioperative management planning and evaluation of new treatments is warranted.

A recent study reported that morbidities following RC are strongly associated with patient-related factors, such as age, performance status, and comorbidities [3]. Although these factors are important measurements of overall health status, they do not enable accurate clinical decisions prior to surgery. For example, reports concerning complications of RC in older individuals often show contradictory and completely opposite results. A substantial number of researchers have reported that RC in octogenarians did not exhibit higher morbidities and could be safely performed [4,5]. However, there are also studies that demonstrate a significant increased risk of complications in old-old and oldest-old patients [6,7]. A recent review of 20 studies of older patients reported significant variation in complication rates: ileus, from 2% to 32%; infection, from 5% to 39% [8]. Taken together, these diverse results suggest that chronological age alone does not sufficiently indicate the worst outcome in older patients treated with RC. Therefore, an improved predictor is needed to assess whether the patient can tolerate such a complex surgical intervention.

Numerous studies have demonstrated that frailty is associated with impaired mobility, disability, poor endurance, and prolonged hospitalization [9-11]. Sarcopenia is a critical physiologic change underlying frailty that can occur as a consequence of aging and malignant disease [12]. The degree of sarcopenia can be quantified using the skeletal muscle index (SMI) from the appearance of muscle on cross-sectional images; this approach is attractive to surgeons because the images are objective and reproducible. We hypothesized that if surgical complications following RC are largely due to impaired resilience and recovery, then a lower SMI could potentially serve as an important prognostic indicator for high-risk surgical patients. In this study, the relationship between lower SMI values and early complications following RC was evaluated by standard reporting methods in a patient cohort.

## Methods

### Patients and surgical intervention

This study was approved by the institutional review board as a minimal-risk study and documented informed consent was obtained from all patients. The medical records of patients who underwent RC at Fudan University Shanghai Cancer Center from July 2007 to March 2013 were retrospectively reviewed. Cases that fulfilled the following criteria were collected: (1) pathological diagnosis of primary BC; (2) preoperative cross-sectional pelvic computed tomography (CT) images were available; (3) RC was performed by experienced surgeons who had completed at least 50 procedures. A total of 247 patients were identified for further analysis. Baseline characteristics, such as age, gender, body mass index (BMI), performance status, Charlson Comobidity Index (CCI), operation time, preoperative hemoglobin, albumin and creatinine levels, and pathological tumor stage and grade were collected.

To determine whether SMI is influenced by the burden of tumor, we established an age-matched non-malignancy control cohort in our study. A total of 204 age-matched population-based controls with no evidence of malignancy were selected from a positron emission topography-CT (PET-CT) screen cohort. Indications for RC were muscle-invasive BC or high-risk non-muscle-invasive BC refractory to transurethral resection and intravesical therapy. Neoadjuvant chemotherapy and/or radiotherapy were not utilized. Indications for orthotopic ileal neobladder were absence of disease at the level of the bladder neck and prostatic urethra, absence of urethral stricture, normal renal function, and patient request for orthotropic bladder substitution. Common contraindications included preoperative stress urinary incontinence, significant comorbidities, and patient unwillingness, unfitness, or inability (by surgeon judgment) to comply with the voiding pattern required by the neobladder.

Second-generation cephalosporin, metronidazole, and elastic compressive stockings were used as prophylaxis agents for infection and thromboembolic events in all patients. Polyethylene glycol solution was routinely administered the day before surgery for mechanical bowel preparation. The majority of patients underwent the procedure under combined general and epidural anesthesia with intrathecal postoperative analgesia.

A standard surgical procedure was performed in all patients, including meticulous pelvic lymphadenectomy with *en bloc* RC as described by Skinner [13]. Orthotopic bladder substitution (according to the Studer ileal neobladder technique [14]) or ileal conduit (according to the Bricker technique [15]) were applied as bladder substitution. The ureteroileal anastomosis was formed using the Nesbit technique [15] with indwelling ureteric stents.

Postoperatively, a nasogastric tube remained in place for all patients and was not removed until the first passage of flatus. Intravenous antibiotics were used preoperatively. The pelvic drainage tube was removed when drainage fluid was less than 30 mL. Ureteric stents were removed 14 days after surgery. Patients with neobladder underwent pouchogram on postoperative day 16 with the urethral catheter removed when there was no significant leakage. At our institution, patients were discharged home after removal of drains, stents, and catheter.

### Assessment of SMI

**Figure 1:**
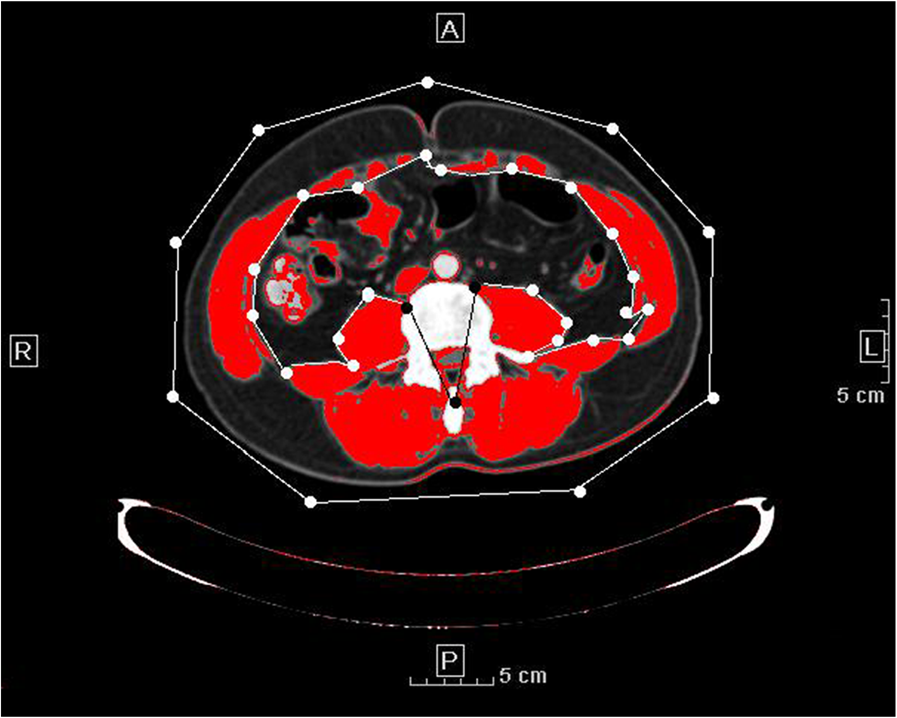
**Computed tomography image showing highlighted areas of skeletal muscle mass (red).** The protocol for measuring areas of skeletal muscle mass is as follows: after converting image type to 8-bit, set scale and double check the scale with the vertical and horizontal ruler. Draw the two regions of interest (ROIs) including the whole section and visceral only, respectively (in the inner line, we excluded spinal cord because some part of spinal cord is at the same density range). Use the XOR function to set the third ROI of the area excluding visceral. Smooth the section twice to reduce noise of image before setting the predefined threshold and measuring the highlighted area.

According to previous reports [16-20], total body skeletal muscle volume can be evaluated by SMI. SMI = skeletal muscle area (SMA)/height^2^. SMA was measured on one cross-sectional scan obtained at the level of the umbilicus (approximately the level of L4 and L5) with the patient in a supine position. This imaging plane was utilized since it strongly correlates with whole body skeletal muscle mass [20] and is easily available in routine pelvic CT scans. As shown in Figure 1, SMA can be calculated automatically by ImageJ software (Version 1.44p, National Institutes of Health, USA) on the basis of predefined Hounsfield unit thresholds (116 to 165). Individuals (FW, YZ) were trained to correctly identify and quantify lumbar vertebrae and the following muscles: rectus abdominus, abdominal (lateral and oblique), psoas, and paraspinal (quadratus lumborum, erector spinae). Intra/interobserver reliability was evaluated and the mean value was used for further analyses.

### Definition and measurement of early complications

Early complications were recorded within 90 days of surgery or during patient hospitalization. In accordance with a recent review of various complications following RC [21], we drafted a checklist for identification of complications from medical records. Complications were defined by clinical and laboratory examinations with/without radiological evaluation. The severity of complications was graded according to Clavien-Dindo classification [22]. Any complication of grade III or higher was defined as a severe complication.

### Statistical analyses

Continuous data were presented as median (range) and category data were presented as proportions. A locally weighted scatterplot smoothing curve and Pearson’s correlation coefficient were used to address the relationship between paired continuous variables. The Student’s t test was performed to test the difference in SMI between categorical variables. Univariate and multivariate logistic regression analyses were performed to evaluate the associations between covariables and outcome. All analyses were performed using R software (version 2.15.2, Institute for Statistics and Mathematics of the WU Wien, Vienna, Austria). The *P* value was two tailed and considered significant when <0.05.

## Results

A total of 208 (84.2%) male patients and 39 (15.8%) female patients were enrolled in this study, mean age was 61.0 year. Detailed demographic and clinicopathological characteristics of the 247 RC patients are shown in Table 1. Table 2 shows the complete list of early postoperative complications. In this cohort, a total of 177 complications occurred in 125 (50.61%) patients. Among these 247 patients, 52 (21.05%) exhibited grade I complications, 54 (21.86%) grade II, 7 (2.83%) grade III, 10 (4.05%) grade IV, and 2 (0.81%) grade V. The rate of severe complication (≥grade III) was 7.69%.

**Table 1:** Demographic and clinicopathological characteristics of the 247 patients included in our study

**Characteristic**	**Measure**	**No. of patients**^**a**^
Sex ratio	M:F	208:39
Age (years)	Median (range)	61.0 (25 to 87)
BMI (kg/m^2^)	Median (range)	22.8 (14.5 to 33.8)
Preoperative Hb (g/L)	Median (range)	135.0 (53 to 167)
Preoperative Alb (g/L)	Mean ± SD	40.9 ± 4.5
Preoperative Cr (μmol/L)	Median (range)	76.0 (35 to 317)
ECOG	0	68 (27.6)
	1	164 (66.4)
	2	13 (5.3)
	3	2 (0.8)
Age-adjusted CCI	≤4	76 (30.8)
	>4, <7	123 (49.8)
	≥7	48 (19,4)
Tumor grade	II	23 (9.3)
	III	224 (90.7)
T stage^b^	Ta and Tis	21 (8.5)
	T1	52 (21.0)
	T2	83 (33.5)
	T3	63 (25.4)
	T4	27 (10.9)
N stage^b^	N0	198 (79.8)
	N1	17 (6.9)
	N2	26 (10.5)
	N3	6 (2.4)
M stage	M0	242 (97.6)
	M1	5 (2.0)
Urinary diversion	Orthotopic neobladder	85 (34.3)
	Ileal conduit	141 (57.1)
	Ureterocutaneostomy	21 (8.5)
Operation time	Median (range)	5.15 (2.08 to 8.58)
Hospital day	Median (range)	20 (9 to 77)

^a^Percentages in parentheses unless indicated otherwise.

^b^T stage and N stage are pathological.

*Alb* albumin, *BMI* body mass index, *CCI* Charlson Comobidity Index, *Cr* creatine, *ECOG* Eastern Cooperative Oncology Group, *Hb* hemoglobin.

**Table 2:** Perioperative complication rates of 247 patients who underwent radical cystectomy (RC)

**Category (n of total**^**a**^**)**	**Complication**	**Frequency**^**b**^
Gastrointestinal	Ileus^c^	37
	Constipation^d^	12
	Gastrointestinal bleeding	1
	Bowel leak	2
	*Clostridium difficile* colitis	2
	Gastric ulcer	2
Infectious	Fever of unknown origin	2
	Urinary tract infection	33
	Sepsis	5
	Pyelonephritis	3
	Gastroenteritis	1
	Cholecystitis	1
	Pelvic abscess	2
Wound	Wound dehiscence	3
	Surgical site infection	12
Genitourinary	Renal failure	1
	Hydronephrosis	5
	Urinary leak	4
	Necrosis of ileal conduit	1
	Parastomal hernia	1
	Testitis	1
Cardiac	Arrhythmia	2
	Myocardial infarction	3
	Acute heart failure	3
Pulmonary	Respiratory distress	1
	Pneumonia	1
Bleeding	Anemia requiring transfusion	2
	Postoperative bleed other than gastrointestinal	1
Thromboembolic	Deep venous thrombosis	3
	Pulmonary embolism	1
Neurological	Peripheral neuropathy	1
	Delirium/agitation	1
Surgical	Vascular injury	1
	Anastamotic bowel leak	2
	Rectum injury	1
Miscellaneous	Lymphatic leak	21
	Other rare complications	Ureter stent broken 2

^a^Reflects the percentage of patients with one or more complication within the category.

^b^Patients experiencing multiple complications of different types are counted more than once.

^c^Ileus is defined as postoperative nausea or vomiting associated with abdominal distension confirmed by radiological examination.

^d^Constipation is defined as inability to have a bowel movement by postoperative day 5 with no signs of ileus or small bowel obstruction. Infectious complications were diagnosed by positive culture. Prolonged lymphatic leak is defined as more than 100 mL drainage output for 2 days starting from postoperative day 3.

The measurement of SMI exhibited low interobserver error (coefficient of variation 0.2% to 3.5%) and low intraobserver error (coefficient of variation 0.9% to 2.9%). A histogram of SMI showed normal distribution (data not shown). Bivariate analysis revealed that SMI was significantly associated with gender (*P* <0.01) but not strongly correlated with age and BMI; in addition, SMI was not associated with operation time (*P* = 0.789). The mean SMI was 42.62 cm^2^/m^2^ in male patients, significantly higher compared to female patients (31.79 cm^2^/m^2^, *P* <0.01). In the age-matched control group, male subjects constituted 80.3% (164 of 204) and the mean age was 60.13 years. In male subjects, SMI in the BC group was decreased significantly compared with the control group (42.62 vs 45.10, *P* = 0.031). However, in female subjects, SMI failed to exhibit a statistically significant difference between the BC group and control group (31.79 vs 32.48, *P* = 0.723).

We evaluated the associations between tested parameters and overall/severe complications (Table 3). According to univariate analysis, the likelihood of overall complications increased dramatically as SMI decreased, if hypoalbuminemia was present and with higher tumor stage (American Joint Committee on Cancer (AJCC) staging >2). In multivariate analysis, hypoalbuminemia and higher tumor stage were independent predictors of morbidity. In univariate analysis, the likelihood of severe complications increased as SMI decreased and age increased (age >70). When other risk factors were adjusted for, aging and lower SMI were found to be independent predictors of severe complications. Each 1 cm^2^/m^2^ increase in SMI decreased the odds of severe morbidity by 4.8%. After dividing patients into four groups according to the value of SMI and BMI, the highest probability of severe complications was observed in cases with low SMI and high BMI (12.2%; nearly three times higher than cases with high SMI and low BMI, 3.3%, *P* = 0.076).

**Table 3:** Univariate and multivariate analysis for evaluation of the associations between tested parameters and short-term complications

**Category**	**Variables**	**Overall**						**Severe (grade ≥3)**					
		**Univariate**			**Multivariate**			**Univariate**			**Multivariate**		
		**OR**	**(95% CI)**	***P *** **value**	**OR**	**(95% CI)**	***P *** **value**	**OR**	**(95% CI)**	***P *** **value**	**OR**	**(95% CI)**	***P *** **value**
Clinical	Gender^a^	0.809	(0.408 to 1.606)	0.545				0.538	(0.120 to 2.407)	0.417			
	Age^a^	1.153	(0.698 to 1.904)	0.578				3.316	(1.068 to 10.298)	0.038	3.376	(1.074 to 10.614)	0.037
	BMI	0.963	(0.887 to 1.044)	0.358				1.012	(0.876 to 1.170)	0.869			
	CCI^a^	1.528	(0.886 to 2.636)	0.128				0.891	(0.332 to 2.394)	0.802			
	ECOG	1.028	(0.468 to 2.257)	0.946				0.746	(0.205 to 2.714)	0.657			
Nutritional	Alb^a^	2.780	(1.041 to 7.424)	0.041	3.628	(1.196 to 11.002)	0.023	1.942	(0.247 to 15.237)	0.528			
	Preoperative Hb^a^	1.912	(0.622 to 5.876)	0.258				1.221	(0.152 to 9.819)	0.851			
	Cr^a^	0.974	(0.406 to 2.337)	0.952				0.488	(0.062 to 3.822)	0.495			
	SMI	0.977	(0.955 to 1.000)	0.045	0.979	(0.954 to 1.004)	0.105	0.951	(0.916 to 0.987)	0.008	0.952	(0.915 to 0.991)	0.017
Tumor specific	AJCC^a^	1.304	(1.004 to 1.693)	0.047	1.289	(0.986 to 1.685)	0.063	0.861	(0.334 to 2.220)	0.758			
	Grade	1.372	(0.577 to 3.258)	0.474				2.157	(0.276 to 16.854)	0.464			
Surgical specific	Diversion^a^	0.871	(0.518 to 1.466)	0.604				1.054	(0.409 to 2.272)	0.913			
	OT	0.980	(0.760 to 1.263)	0.876				0.826	(0.530 to 1.287)	0.397			

^a^Category variables: male patients versus female patients; diversion: orthotopic neobladder versus non-orthotopic neobladder; cut points are indicated as follows: age >70; preoperative Hb female patients <110 g/L or male patients <120 g/L; Alb <35 g/L; creatine (Cr) >110; AJCC >2 or otherwise, CCI > 7.

*AJCC* American Joint Committee on Cancer, *Alb* albumin, *BMI* body mass index, *CCI* Charlson Comobidity Index, *Cr* creatine, *ECOG* Eastern Cooperative Oncology Group, *Hb* hemoglobin, *OT* operation time (h), *SMI* skeletal muscle index.

## Discussion

Although surgical techniques have improved in recent years, morbidity remains high for operational trauma. In centers of excellence, morbidity ranges from 28% to 50% [3,23,24]. Using standardized complication reporting methodology, the prevalence of overall mortality was 50.61% in our study, which is in concordance with previous reports. Predictors of complications are needed to enable improved decision making, assessment of eligibility for clinical trial, and provide performance benchmarks. Muscle wasting is recognized as a physical condition in the aged population and is an emerging concern in patients with malignant disease. A recent report indicates a positive link between lower SMI and short-term postoperative morbidity [25]. To the best of our knowledge, this study reports the relationship between lower SMI and perioperative morbidity of RC for the first time. In multivariate analysis, SMI and aging are the two independent predicting factors of severe complications. We reviewed the medical records and found that senior patients more frequently experienced heart disease or deep vein thrombosis than younger patients. These factors may explain why senior age is correlated with severe complications but not overall complications, and this is supported by several previous reports [6,7]. SMI exhibited a negative relationship with short-term morbidity, whereas a positive relationship was found between BMI and short-term morbidity; high BMI tended to be associated with an increased adverse event rate. In fact, Svatek previously investigated this in RC patients and arrived at the same conclusion (OR = 1.16, *P* <0.001) [3]. It is understood that muscle wasting is not necessarily associated with fat loss [26]. Covered by a mantle of adipose tissue, muscle wasting is often an occult condition and neglected by surgeons. In this study, we discovered that patients with lower SMI as well as obesity were at the highest risk of early complications. Therefore, these findings suggest that SMI, which is quantified by the appearance of muscle on cross-sectional imaging, could be valuable in risk stratification and offer useful information in clinical decision making, especially in patients with BC under consideration for radical cystectomy.

Lower SMI is frequently observed in cancer patients [27,28]. Compared to cachexia, lower SMI is more prevalent in the early stages of disease. The reason for exacerbation of lower SMI is multifactorial. First, some risk factors of cancer are also strongly associated with lower SMI. It has been independently reported that aging and smoking are risk factors of both bladder cancer and lower SMI [29-32]. Second, alteration of metabolism is another hallmark of cancer. Cancer cells are highly metabolic, leading to diminished homeostatic reserves from release of endogenous transmitters and changes in inflammatory markers and other mediators [33,34]. Third, the treatment of cancer is commonly accompanied by adverse events that may cause malnutrition in patients. For example, reduced nutritional intake (for example, anorexia) can be exacerbated by the side effects of anticancer therapies [35].

Lower SMI is a modifiable prognostic factor of BC patients undergoing RC. Malafarina *et al*. systemically reviewed intervention of muscle wasting and concluded that nutritional supplementation is an effective treatment in older patients [36]. Nutritional supplementation could potentially lead to less morbidity and faster recovery for BC patients undergoing RC, especially in patients with lower SMI.

Assessment of SMI is convenient in clinical practice because only a single slice of CT is needed. As these images are routinely available in clinical records, no extra testing is usually required. Moreover, a surgeon can measure the lumbar skeletal muscle within 5 minutes; thus, it is an affordable and efficient method that is specific, accurate, and sensitive to change.

Several limitations should be acknowledged in this pilot study. Though the number of cases who experienced severe complications was limited, validation is needed in a larger population. Since this was a single center analysis, further prospective studies in other centers are also needed for validation. Our study included patients who underwent radical cystectomy with available perioperative CT scans; thus, there may be a selection bias regarding patients excluded without available CT scans. Because the ethnic population was limited to Asians, the distribution of lumbar skeletal muscle index differs from the Western population [37,38]. Therefore, the study should be validated in other ethnic populations as well.

## Conclusions

SMI is a specific tool easily acquired in routine clinical practice. Lower SMI is frequently observed in bladder cancer patients undergoing RC and is shown to be strongly associated with early complications following surgery.

## Competing interests

The authors declare that they have no competing interests.

## Authors’ contribution

FW, ZY and DY designed the study and FW, ZY drafted the manuscript. CG participated in the manuscript drafting and revising. XY, YS, BD, SZ, HZ provided the clinical data of patients who underwent radcial cystectomy. JC provide the clinical data of non-malignant control. All authors read and approved the final manuscript.
